# What influences the perceived access to healthcare services? An investigation using Structural Equation Modeling

**DOI:** 10.3389/fpubh.2026.1844139

**Published:** 2026-06-04

**Authors:** Murat Topbas, Irem Dilaver, Serdar Karakullukcu, Fusun Yalcin, Husniye Ebru Colak, Elif Bahcekapili, Nazim Ercument Beyhun, Mehmet Topsakal, Yavuz Cakiroglu, Erdem Sen

**Affiliations:** 1 Department of Public Health, Faculty of Medicine, Karadeniz Technical University, Trabzon, Türkiye; 2 Department of Mathematics, Faculty of Science, Akdeniz University, Antalya, Türkiye; 3 Division of Land Management, Department of Geomatics Engineering, Faculty of Engineering, Karadeniz Technical University, Trabzon, Türkiye; 4 Trabzon Provincial Health Directorate, Trabzon, Türkiye

**Keywords:** access to health care, health literacy, health-seeking behavior, socioeconomic status, spatial analyses, Structural Equation Modeling

## Abstract

**Background:**

Access to healthcare is a fundamental determinant of public health sustainability. Current literature on healthcare access tends to focus largely on vulnerable groups and objective indicators where access is defined through service utilization. When the literature on “perceived access to healthcare” is examined, it is observed that perceived access is generally treated as a determining factor explaining health outcomes or behaviors. However, the investigation of the socioeconomic, self-rated health, health literacy and spatial features that shape this perception itself within a holistic framework remains a less focused area in the literature. Methodologically, perceived access to healthcare is often defined with a limited number of variables or specifically within particular disease groups. Especially in regions such as Trabzon, where rugged topography and geographical barriers are intertwined with socio-cultural dynamics, analyzing the factors shaping the individual’s perception of access within a comprehensive framework will offer a significant contribution to the literature. This study aims to integrate multidimensional factors -including socioeconomic status (SES), self-rated health, health literacy, health-seeking behavior, and travel time-into a comprehensive structural framework for the first time in the literature; to validate their effects through Structural Equation Modeling (SEM), and to uncover the spatial dependence patterns in access.

**Methods:**

This reearch was conducted with 1,491 adults. Data were analyzed using Structural Equation Modeling (SEM) to evaluate multidimensional pathways, and geospatial analysis was performed to determine spatial dependence patterns.

**Results:**

The results of the research demonstrate that health literacy and professional health-seeking behavior are the primary elements strengthening the perception of access, whereas online health-seeking and increased travel time weaken this perception. One of the most original findings of the study is that the direct negative effect of SES on access is balanced through health literacy; this indicates that cognitive capacity can mitigate socioeconomic disadvantages. Spatial models confirm that while the general perceived access to healthcare is shaped by personal factors, the “accessibility” dimension exhibits a spatial dependence tied to local topography within a 1-km radius.

**Conclusion:**

Consequently, these findings scientifically support that health planning should shift from macro-scale strategies toward micro-spatial interventions aimed at minimizing physical barriers at the neighborhood and street levels. Integrating approaches that improve health literacy with interventions that minimize travel barriers will play a fundamental role in reducing health inequalities.

## 1 Introduction

Access to healthcare is recognized as a key determinant in the processes of measuring and evaluating the performance of modern healthcare systems and in ensuring the sustainability of public health. Historically, the concept was confined to supply-side variables, such as the geographic location of healthcare facilities or the cost of healthcare services. Research in the literature evaluated advanced spatial analysis techniques performed with GIS-based systems, using such as average nearest neighbor analysis, equal weighted overlay approaches, Euclidean distance, and kernel density estimation, which primarily affect access to healthcare services (1, 2). However, in the modern approach, it has evolved into a multidimensional framework encompassing the individual’s experience of accessing healthcare and the socio-cultural barriers shaping that experience. The “five A’s” framework, developed by Penchansky and Thomas (3), addresses access to the healthcare system as the alignment between the patient and the healthcare system. This concept is evaluated across five dimensions: availability, accessibility, accommodation, affordability, and acceptability. This model enables the evaluation of the concept of access from not only a supply-side perspective but also from the perspective of the patient’s experience. On the other hand, Andersen’s Behavioral Model elucidates these elements within the framework of predisposing factors, encompassing demographic and sociocultural characteristics such as age, gender, educational level, and cultural beliefs that determine access and utilization of services. Enabling factors include resources that facilitate access to services, including income level, health insurance coverage, and physical access to healthcare facilities. Need factors encompass the individual’s self rated health status or clinically determined health needs (4, 5). Levesque and colleagues’ access model employs a dynamic and multidimensional approach by evaluating both supply and demand dimensions concomitantly (6, 7). These comprehensive definitions and conceptual frameworks demonstrate that access to health services is not a concept that can be evaluated in a one-dimensional manner; rather, it is a multidimensional and dynamic process shaped by the mutual interaction of individual characteristics, health system capacity, socioeconomic conditions, and cultural factors.

International and national reports evaluate access to healthcare through variables such as the inclusiveness of healthcare services, the capacity of healthcare services, and unmet health needs. The World Health Organization (WHO) and the World Bank’s 2023 Universal Health Coverage (UHC) Global Monitoring Report indicates that, as of 2021, approximately 4.5 billion people worldwide—roughly half the global population-lacked full access to essential health services; and that in 2019, approximately 2 billion people faced financial hardship due to out-of-pocket health expenditures. The report also highlights that limited progress was made in the Universal Health Coverage Index between 2015 and 2021, with inequalities becoming more pronounced, particularly in low- and middle-income countries (8). In the period following the pandemic, the waiting times for elective care in numerous OECD countries persist at levels that exceed pre-pandemic benchmarks (9). Conversely, the European Union’s 2023 Synthesis Report underscores an escalation in the burden on the healthcare workforce and a heightened prevalence of access challenges, particularly in rural regions (10). According to OECD indicators and national health statistics from 2023, while health services in Türkiye achieve near-universal population coverage, there remain specific areas for improvement regarding the quality of care and the system’s capacity (9, 11–13). The satisfaction rate regarding access to health services is 53%, which is significantly below the OECD average. Financial coverage and out-of-pocket expenditures are in line with OECD levels. Additionally, while the number of physicians (2.2), nurses (2.8), and hospital beds (3.0) per 1,000 people in Türkiye is increasing, these figures remain below the OECD average (9). Beyond these numerical and capacity-oriented data, it is essential to consider the structural and institutional framework of the system to fully comprehend the dynamics of healthcare access in Türkiye. Turkish Healthcare System, which is designed to provide comprehensive and universal coverage. Primary care services are extensively distributed nationwide through the Family Medicine System, ensuring that every citizen is formally registered with a specific family physician (13). A defining characteristic of the Turkish model is the absence of a compulsory referral chain; patients possess the autonomy to bypass primary care and directly access secondary or tertiary levels of medical intervention. Consequently, individuals can freely utilize emergency services and outpatient polyclinics at both public and private hospitals, exercising their legal right to select their own physician for diagnostic and therapeutic processes (14, 15). This systemic flexibility is strategically intended to maximize service availability and patient agency.

Identifying the level of access to healthcare in a society, as well as its determinants, is critical not only for measuring the efficiency of a system but also for ensuring social justice and public health security. In current literature, access to healthcare and its determinants have been evaluated through various approaches. A significant portion of original research assessing healthcare access and its influencing factors focuses on vulnerable groups such as women (16), the older adults (17, 18), rural residents (19, 20), migrants (21, 22), and individuals with specific health conditions (18, 23, 24). Research that addresses the determinants of healthcare access more comprehensively is commonly in the form of reviews or systematic reviews (25, 26). Additionally, some studies have focused on identifying barriers to healthcare access (27–30).

Most of these studies in the literature tend to define access to healthcare through objective and measurable indicators, such as utilization of health institutions. However, the extent to which these services are perceived as ‘accessible’ by the individual is not sufficiently evaluated. At this point, the concept of ‘perceived access,’ which centers on how an individual interprets the health system and evaluates its ease of use, has gained critical importance in the literature. Regarding perceived access to healthcare, research investigating local amenities such as public transportation and parking areas has shown that perceived access is more useful in understanding and predicting human behavior than physical accessibility (31, 32).

Research indicates that a significant portion of studies on perceived access to healthcare focuses on its impact on dependent variables such as health outcomes or health behaviors (33–35). Some research has specifically focused on the perceived access of individuals with certain diseases (35–38). When evaluated methodologically, different approaches have been used to determine perceived access to healthcare. While some studies assess perceived access through a single or a limited number of questions, such as the ease of access or the ability to reach needed health services (33, 38, 39), others have evaluated spatial perception in healthcare access (34, 40). Some studies, emphasizing that access is a dynamic and multidimensional structure, have utilized scales that reflect this complexity (35, 37). Considering the complex nature of the concept of perceived access to healthcare, it is thought that literature generally handles perceived access within a narrower scope and examines its interaction with a limited number of variables.

The world and its settlements are not a flat tray. Geographical and topographical conditions cause people to live at altitudes significantly different from sea level. The urban and rural settlement areas of countries create serious geography-based differences, which can lead to economic, social, and cultural variations among individuals and society. Similarly, the provision of healthcare services is shaped by the geographical, topographical, economic, and social characteristics of a country. In the case of Turkey, in cities like Trabzon where geographical barriers (slope, rugged terrain, etc.) and socio-cultural dynamics are decisive factors in healthcare access, there is a need to examine the factors shaping individual perception through a holistic model.

The aim of this study is to holistically integrate multidimensional factors including socioeconomic status, self rated health, health literacy, health seeking behavior, and travel time—for the first time in the literature; to validate their effects on perceived access to healthcare through Structural Equation Modeling (SEM), and to uncover the spatial dependence patterns in perceived access.

## 2 Materials and methods

### 2.1 Methodological workflow

**Figure 1:**
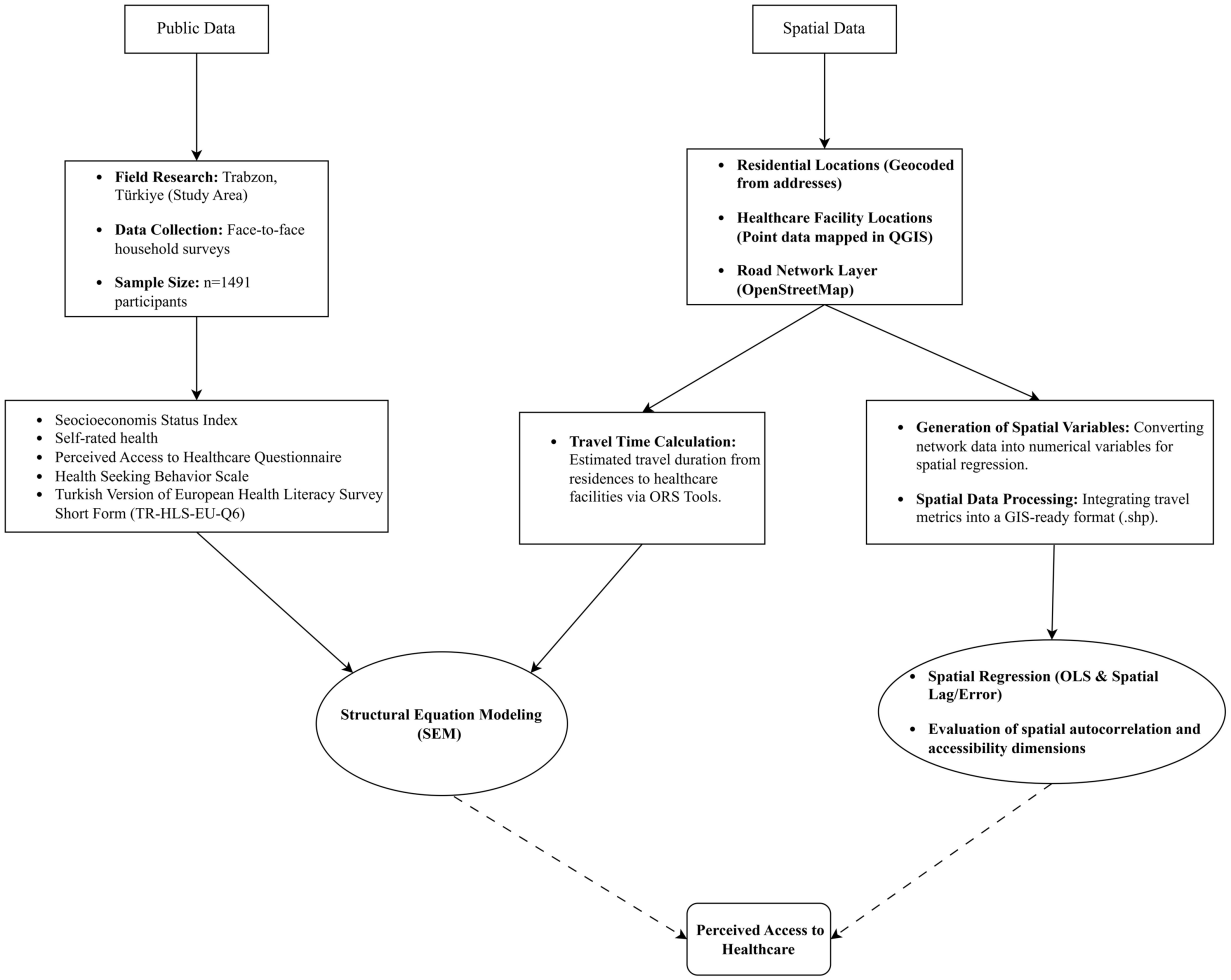
Flowchart of the integrated methodological framework according to data species.

The present study employs an integrated approach combining large-scale household survey data with geospatial network analysis. The systematic workflow of the research -covering data collection, spatial processing, and advanced statistical modeling—is illustrated in Figure 1.

### 2.2 Study area

The present research was conducted in Trabzon, a province situated within the Eastern Black Sea Region of Türkiye. The topographical landscape of Trabzon is defined by mountainous and rugged terrain, which introduces significant geographical variances in the delivery and attainment of healthcare services (41). These variations are particularly pronounced between the coastal and inland areas, which are separated by these geographical differences. Trabzon’s sociodemographic characteristics are shaped by migration and urbanization, resulting in a population with high proportions of both young and older adults individuals. The province exhibits a notable aging demographic, characterized by a substantial proportion of older adults residents and a high old-age dependency ratio, while concurrently experiencing low fertility rates. This situation underscores the province’s vulnerability in terms of health (13, 42). In terms of healthcare infrastructure, Trabzon functions as a regional healthcare hub, featuring a university hospital, a city hospital currently under construction, and various secondary and tertiary healthcare facilities. These facilities experience a significant influx of patients from neighboring provinces and abroad, which increases the workload per physician and nurse, prolongs waiting times, and strains existing capacity. However, the relatively high number of physicians and hospital beds in comparison to the national average is regarded as one of the province’s strengths (13). The unique characteristics of Trabzon, including its geography, demographics, and infrastructure, position it as a suitable and distinctive field for a multidimensional analysis of the determinants of access to healthcare services.

### 2.3 Study population and sampling

The study population consists of individuals aged 18 and over residing in Trabzon, Türkiye. According to the 2025 Address-Based Population Registration System data, the adult population of Trabzon is 640,506, comprising 315,029 males and 325,477 females (42). To represent this population effectively, the study sample was determined using a stratified random sampling method.

**Figure 2:**
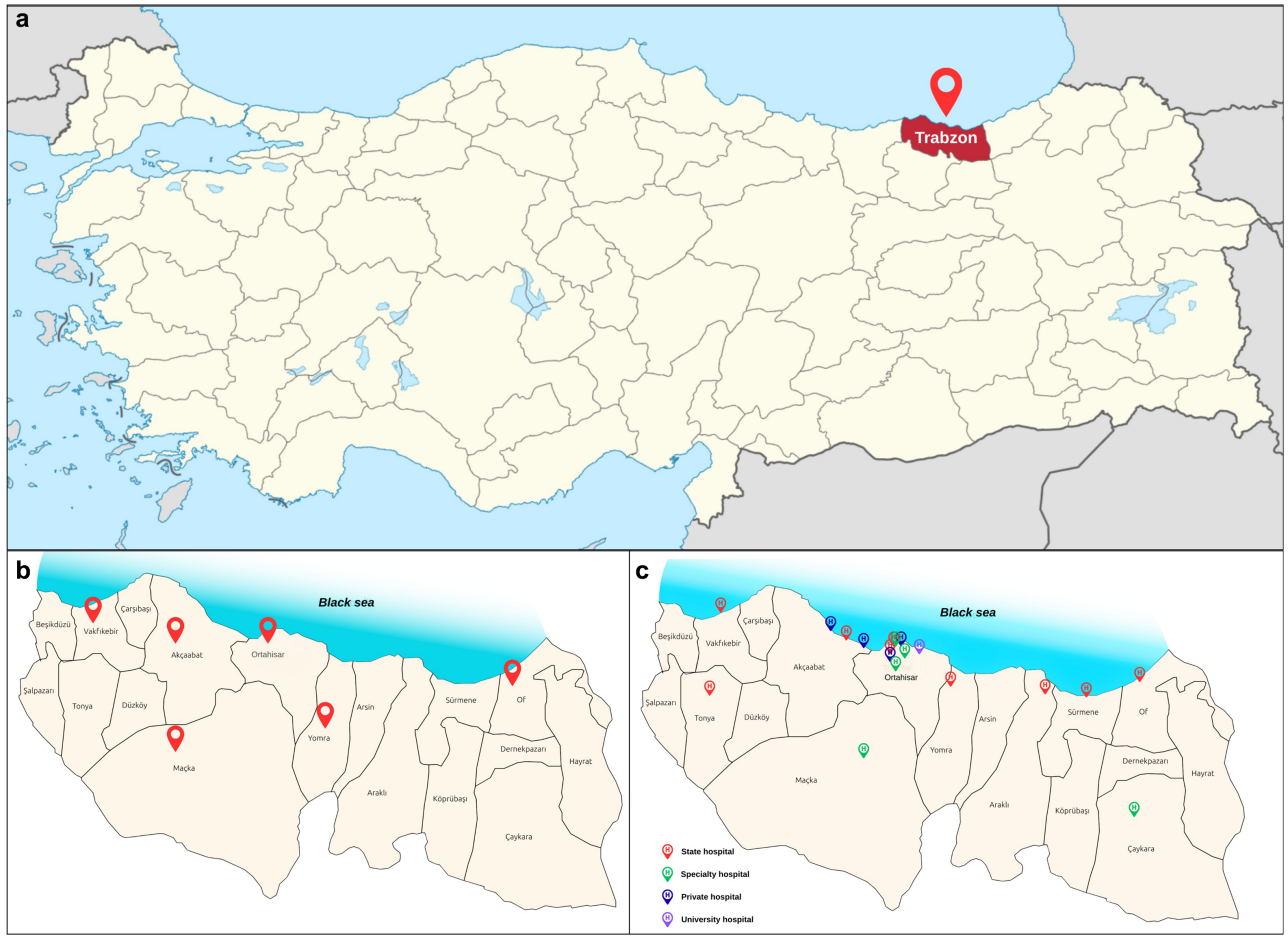
Cope of the health care access research in Trabzon. **(a)** Location of Trabzon province within the map of Türkiye; **(b)** study regions (districts) where survey data were collected; **(c)** spatial distribution of hospitals in Trabzon province. Panel **(a)** adapted from “Trabzon in Turkey.svg” by TUBS, Wikimedia Commons, CC BY-SA 3.0. Adapted by the authors.

**Figure 3:**
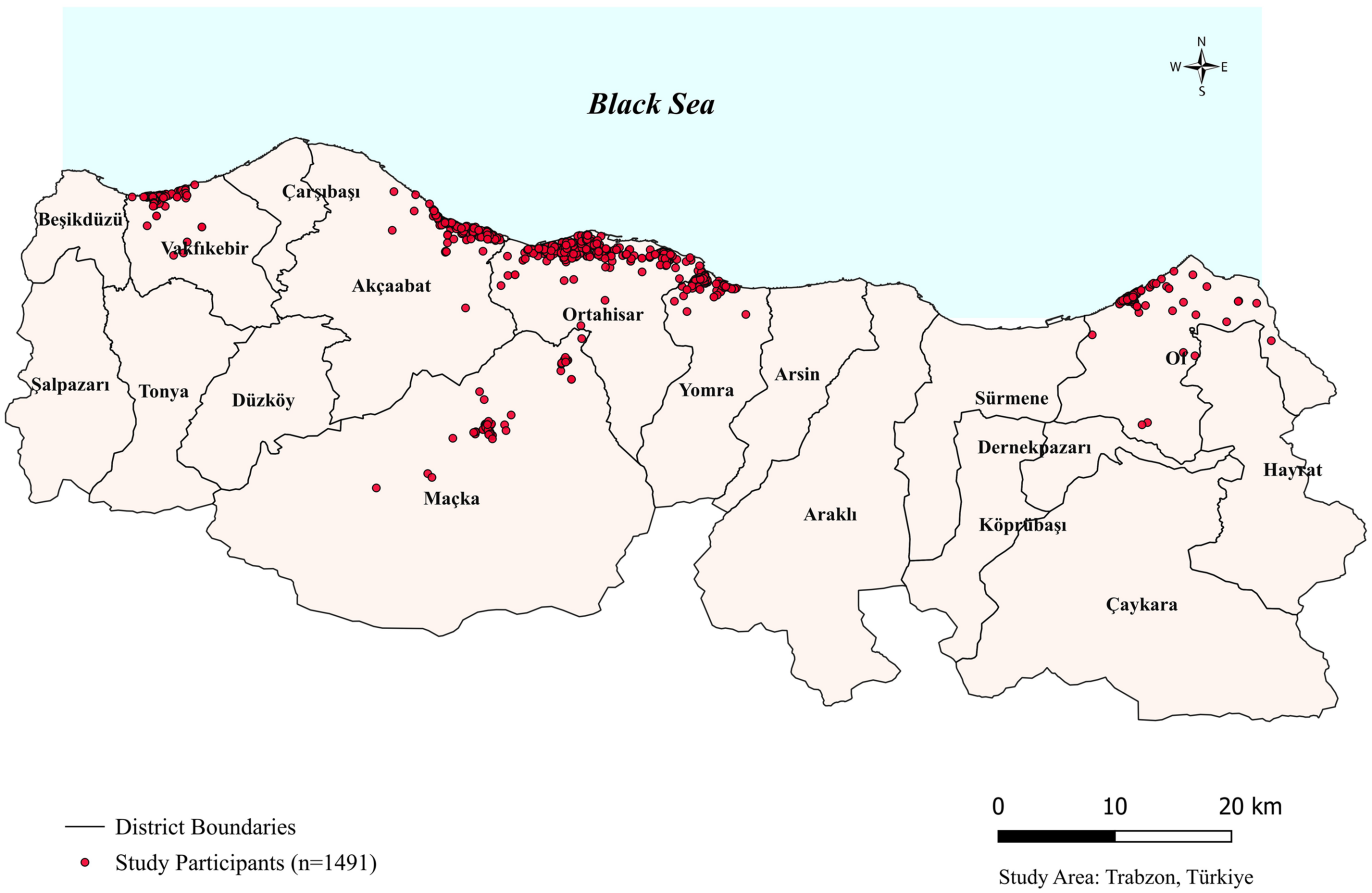
Spatial distribution of participants across Trabzon districts (*n*=1491).

To fully reflect the geographical and demographic heterogeneity of the region, six districts representing the southern (1 district), eastern (2 districts), western (2 districts), and central (1 district) regions of the province were included as strata. Proportional stratification based on age and gender variables within these selected districts was implemented to enhance the representativeness of the sample and the reliability of the findings. The geographical location of Trabzon within Türkiye (Figure 2a), the specific districts where data collection was conducted (Figure 2b), and the spatial distribution of hospitals in Trabzon (Figure 2c) are presented in Figure 2 and spatial distribution of participants shown in Figure 3.

The sample size (*N* = 1,491) provides robust statistical power for the structural model. This volume adheres to the recommended 10:1 participant-to-parameter ratio ensuring stable parameter estimation and enhancing the reliability of the hypothesized mediation pathways (43, 44).

### 2.4 Data collection and instruments

Data were collected using a structured survey instrument, adapted from extant literature evaluating the determinants of health care access and refined through expert consultation to ensure its suitability for the research objectives. Following a preparatory training session for the enumerators, data were collected through face-to-face interviews conducted at the participants’ households. The data collection instrument comprises six primary sections:

*Section 1. Sociodemographic and personal characteristics:* this section elicited information on participants’ age, gender, marital status, educational attainment, occupation, employment status, and total monthly household income, as well as household composition.

*Section 2. Health status characteristics:* this section assessed participants’ self-rated health, the presence of chronic diseases, and the regular use of medications.

*Section 3. Geographical and transportation characteristics:* this section recorded participants’ residential addresses, their registered Family Health Centers (FHCs), the healthcare institution most frequently visited within the past year, and the primary mode of transportation used to access these facilities.

*Section 4. Turkish version of European Health Literacy Survey Short Form (TR-HLS-EU-Q6):* The 6-item European Health Literacy Survey Short Form (HLS-EU-Q6) was employed to evaluate participants’ health literacy levels. This instrument is a condensed version of the original 47-item scale developed by Sørensen et al. (45) within the framework of the European Health Literacy Project (HLS-EU, 2009–2012). The HLS-EU-Q6 specifically measures the individual’s capacity to access, comprehend, appraise, and apply health-related information through six targeted items. Responses are recorded on a 4-point Likert-type scale, ranging from 1 (very difficult) to 4 (very easy). The Turkish adaptation and psychometric validation of the short form were conducted by Yeşildal (46), who reported a Cronbach’s alpha coefficient of 0.82. Mean scores, ranging from 1 to 4. Yeşildal’s (46) study confirmed the TR-HLS-EU-Q6 as a pragmatic and reliable tool for rapid health literacy assessment within the Turkish population.

*Section 5. Health-seeking behavior scale:* the health-seeking behavior scale, developed by Kıraç and Öztürk (47), was used to determine the pathways individuals follow when encountering health problems. The initial development study involved 401 participants aged 18 and older; subsequent Exploratory Factor Analysis (EFA) and Confirmatory Factor Analysis (CFA) revealed a 12-item, 3-factor structure. The scale encompasses three sub-dimensions: online health-search behavior (6 items), professional health-search behavior (3 items), and traditional health-search behavior (3 items). Items are rated on a 5-point Likert scale (1 = strongly disagree to 5 = strongly agree). Reliability analyses yielded Cronbach’s alpha coefficients of 0.726, 0.720, and 0.736 for the respective sub-dimensions, with an overall scale alpha of 0.755, indicating robust internal consistency. Higher mean scores in a specific sub-dimension reflect a greater tendency toward that particular health-seeking mode, making the scale particularly effective for community-based research (47).

*Section 6. Perceived access to healthcare questionnaire:* The perceived access to healthcare questionnaire, originally developed by Hoseini-Esfidarjani et al. (48) and later adapted into Turkish by Yılmaz et al. (49), was utilized to assess participants’ experiences regarding healthcare accessibility. In the Turkish validation study, the scale demonstrated high reliability, with a total Cronbach’s alpha of 0.92 and sub-dimension alphas ranging from 0.81 to 0.90. The instrument consists of 23 items categorized into four factors: accessibility (4 items), acceptability (8 items), accommodation (8 items), and affordability (3 items). All items are evaluated using a 5-point Likert scale (1 = strongly disagree to 5 = strongly agree). Scoring is performed by dividing the sum of the sub-dimension items by the number of items within that factor, while the total access score is derived by dividing the total scale sum by 23. Elevated scores signify a more positive perception of access to healthcare services (49).

### 2.5 Theoretical framework and model specification

The theoretical foundation of this research is constructed by synthesizing Andersen’s (5) Behavioral Model of Health Services Use with Penchansky and Thomas’s (3) Theory of Access. This integrated framework designates “perceived access to healthcare” as the primary outcome variable, while systematically categorizing independent predictors into three functional domains: predisposing (Health Literacy and Health-Seeking Behaviors), enabling (socioeconomic status and travel time), and need factors (self-rated health).

**Figure 4:**
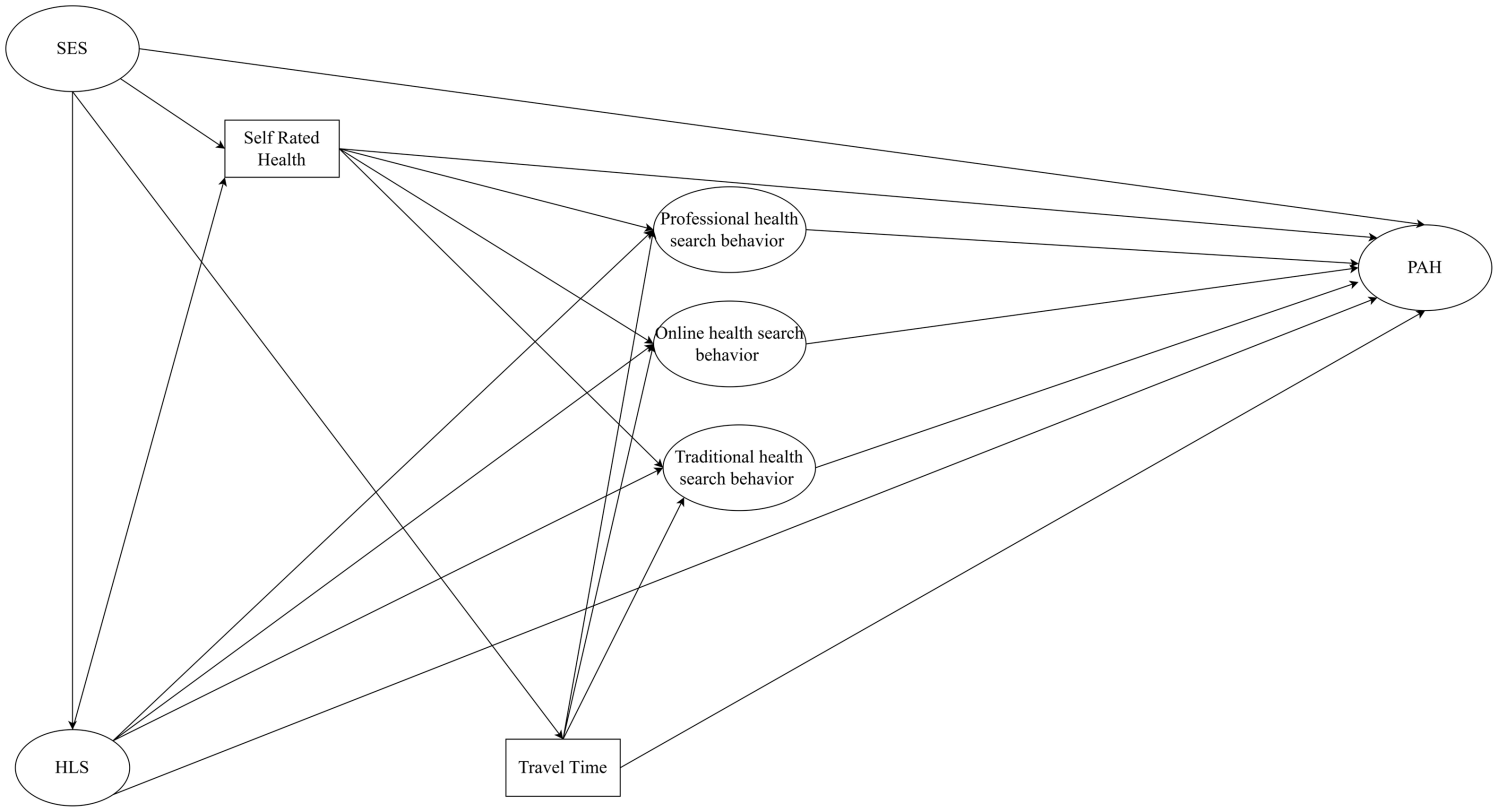
Conceptual framework of the Structural Equation Modeling (SEM).

The structural integrity of the analytical framework is presented in Figure 4. In accordance with standard SEM notations, latent constructs are represented by ellipses, while observed variables are depicted in rectangular boxes.

#### 2.5.1 Measurement and operationalization of variables

To ensure measurement transparency and address the specific psychometric properties of the model, the variables were operationalized as follows:

*The socioeconomic status (SES) index (latent variable):* Constructed by the researchers as a composite measure comprising education level, monthly household income, and occupational status. Inspired by the hierarchical framework developed by Kuppuswamy, the index was adapted by the researchers to align with the current socioeconomic indicators of Türkiye (50, 51). Income levels were categorized relative to the official ‘hunger threshold’ (HT) as reported by the Confederation of Turkish Trade Unions (TÜRK-İŞ, 2025) (52). This threshold represents the minimum monthly food expenditure for a family of four. While household size and type were initially considered, they were excluded following preliminary Confirmatory Factor Analysis (CFA) due to insufficient factor loadings and a lack of significant contribution to the construct’s variance. Occupational status was classified according to the International Standard Classification of Occupations (ISCO-08), ranging from unemployed/retired individuals to high-level managers and professionals (53). The structural validity of this researcher-derived index was confirmed through Confirmatory Factor Analysis (CFA). Detailed scoring and components of the SES index are presented in Table 1.

**Table 1:** Components and scoring of the SES index.

Score	Education level	Monthly household income	Occupational status (ISCO-08)
1	Primary school	<1× HT	Unemployed
2	Primary school graduate	≥1× HT, <1.5× HT	Retired
3	Middle school graduate	≥1.5× HT, <2× HT	Plant and machine operators and assemblers; Elementary occupations
4	High school graduate	≥2× HT, <3× HT	Skilled agricultural, forestry and fishery workers; craft and related trades workers
5	University graduate	≥3× HT	Clerical support workers; service and sales workers; technicians and associate professionals
6	Post-graduate degree	–	Managers; professionals

HT, hunger threshold, ISCO-08: International Standard Classification of Occupations.

*Health literacy and professional, online, traditional health search behaviors (latent* var*iables):* measured using the validated TR-HLS-EU-Q6 and the health-seeking behavior scale, respectively. Their predefined factor structures were verified through CFA to ensure suitability for the study population. Beyond their direct influence, these factors are also evaluated for their potential mediating roles in the complex relationship between socioeconomic status and access (46, 47, 54).

*Travel time (observed variable):* travel time was computed using ORS Tools (OpenRouteService), calculating the time from each participant’s residential address to their most frequently visited healthcare facility based on their self-reported mode of transportation. For participants reporting the use of public transit (minibuses or city buses), the ‘driving-car’ mode was utilized as a representative proxy. This approach was adopted to maintain methodological consistency, as high-resolution, real-time public transit data and standardized stop frequencies were unavailable for the diverse residential locations. Furthermore, this proxy aligns with the urban infrastructure of Trabzon, where the absence of rail-based or segregated transit systems necessitates that all vehicles share the same road network and traffic conditions. In the structural model, travel time was operationalized as a categorical variable using a 15-min threshold. This specific cutoff was selected to align with the ‘15-min city’ framework, which identifies this duration as a critical benchmark for high-level local accessibility, including access to healthcare services (55, 56). Furthermore, considering the distinctive topographical features of Trabzon—characterized by steep slopes and rugged terrain—and the specific distribution of the local data, this threshold was utilized to maintain the necessary statistical sensitivity for the SEM analysis.

*Self-rated health (observed* var*iable):* it was measured using a single-item question: “In general, how would you rate your health?” Responses were recorded on a 5-point Likert scale, ranging from 1 (Very Poor) to 5 (Very Good). This subjective assessment is widely utilized in public health research as a robust and comprehensive indicator of an individual’s overall health status and their perceived need for healthcare services.

*Control* var*iables:* Age and gender were included as exogenous observed control variables to mitigate potential confounding effects.

#### 2.5.2 Mediation mechanisms and structural model

The indirect effects within the model were tested through an integrated structural model designed to encompass both parallel and serial mediation mechanisms. Within this context, the hierarchical flow between variables was specified across four primary layers:

1. SES-driven paths: the effects of SES on the perception of access through HLS, self-rated health, and travel time.
2. Health literacy (HLS)-driven serial paths: chain pathways where HLS serves as a precursor to self-rated health, which in turn shapes PAH through Health-Seeking Behaviors.
3. Self-rated health-driven paths: the impact of self rated health on perceived access to healthcare via health-seeking behavior.
4. Travel time-driven paths: the impact of travel time factors on perceived access to healthcare through their interaction with behavioral preferences.

### 2.6 Data analysis

#### 2.6.1 Descriptive statistics and comparative analyses

The dataset obtained from the research was subjected to descriptive and inferential statistical evaluations utilizing IBM SPSS Statistics 23.0. Descriptive characteristics for categorical variables were reported as frequencies and percentages, while continuous variables were summarized through means, standard deviations, and minimum-maximum values to provide a comprehensive overview of the sample. The normality of the data distributions was assessed using the Kolmogorov–Smirnov test. Given that the normality assumptions were not met across the numerical variables, the Kruskal–Wallis *H* test was utilized for comparative evaluations. Throughout all statistical procedures, the threshold for significance was strictly maintained at a *p*-value of less than 0.05.

#### 2.6.2 Structural Equation Modeling

Data analysis was conducted using IBM SPSS AMOS (Version 24.0) following a two-step approach. First, the measurement validity of the latent constructs was verified through Confirmatory Factor Analysis (CFA). The fit indices indicated that all scales demonstrated acceptable to good fit with the data: perceived access to healthcare questionnaire [Comparative Fit Index (CFI) = 0.921, Root Mean Square Error of Approximation (RMSEA) = 0.067], TR-HLS-EU-Q6 (CFI = 0.978, RMSEA = 0.057), and health-seeking behavior scale (CFI = 0.959, RMSEA = 0.051). Detailed results of the measurement models, including individual item-factor loadings and significance levels, are presented in the Supplementary material. For the SES Index, a CFA was conducted where standardized factor loadings ranged from 0.61 to 0.73. The reliability was established through Composite Reliability (CR) of 0.72 and a Cronbach’s alpha (*α*) of 0.692. Although the alpha value is slightly below the 0.70 threshold, it is considered acceptable for a three-item composite measure, and the CR value further confirms the internal consistency of the construct (57).

Second, Structural Equation Modeling (SEM) was employed to test the hypothesized relationships. In this framework, socioeconomic status (SES) was established as the primary exogenous latent construct, while age and gender were integrated as control variables. Health literacy, health-seeking behaviors, self-rated health, and travel time served as mediating variables leading to the focal endogenous construct, perceived access to healthcare (PAH).

Model adequacy was evaluated using the Chi-square/degree of freedom (*χ*^2^/df) ratio, the Comparative Fit Index (CFI) (>0.90), the Tucker–Lewis Index (TLI) (>0.90), the Goodness of Fit Index (GFI) (>0.90), the Root Mean Square Error of Approximation (RMSEA) (<0.08), and the Standardized Root Mean Square Residual (SRMR) (<0.08) indices. To unravel the complex indirect pathways, a mediation analysis was conducted using the bootstrapping method (5,000 resamples) with 95% bias-corrected confidence intervals (BC CI) to determine the statistical significance of the mediating roles played by mobility, literacy, and behavioral patterns.

#### 2.6.3 Generation of maps and spatial modeling

The spatial representation of the research data was executed by transforming participant residential information into a discrete point dataset. Utilizing the address details from the participant database, a geocoding process was performed to convert each survey respondent into a geographically referenced point feature. Administrative boundary datasets, encompassing the provincial and district borders of Trabzon, served as the fundamental spatial framework for the analyses, supplemented by a point data layer representing district centers. To ensure spatial integrity, these datasets were reprojected into a unified coordinate system and integrated within the GIS environment, facilitating the execution of spatial analyses within the defined study area. All geospatial operations were conducted using QGIS (version 3.44) software and spatial data explorations were conducted using GeoDa 1.22 software. Within this framework, all point and linear datasets were maintained in vector format. Real-world travel times and road network distances were calculated using the OpenRouteService (ORS) Tools plugin, integrated within the QGIS environment. To provide geographic context and verify the network structure, the OpenStreetMap (OSM) was utilized as an open-source basemap.

To examine the dynamics of perceived access to healthcare and its four sub-dimensions, OLS and Spatial Lag Models (SLM) were employed; however, for the accessibility sub-dimension, the Spatial Error Model (SEM) was preferred at the 1 km threshold as diagnostic tests indicated spatial dependence in the error terms. Diagnostic tests were conducted to ensure the structural integrity of the models. The Condition Number was calculated as 36.93 across all OLS models. This value indicates that multicollinearity among the independent variables remains within a manageable range, suggesting no significant instability in the coefficient estimates. The Jarque–Bera test results (*p* < 0.001) revealed that the error terms are not normally distributed. Furthermore, the significance of the Breusch–Pagan and Koenker–Bassett tests (*p* < 0.05) indicates the presence of heteroskedasticity. Consequently, a cautious approach was adopted in interpreting the spatial estimators, necessitating a focus on robust standard errors and model stability.

To determine the scope of spatial interaction and its sensitivity to scale, the analyses were conducted using multiple distance bands (1 km, 3 km, and 10 km) and neighborhood criteria based on queen contiguity analysis. This multi-scale approach was employed to identify the specific geographic level at which perceived access to healthcare clusters and to minimize potential biases arising from the selection of the spatial weight matrix.

### 2.7 Ethical approval

This study was approved by the Scientific Research Ethics Committee of the Faculty of Medicine at Karadeniz Technical University (approval no. 2025/287).

## 3 Results

### 3.1 Socio-demographic profiles and descriptive statistics

Of the participants, 758 (50.8%) were female and 1,013 (67.9%) were married. The mean age was 44.1 ± 15.8 years. In terms of residency, 567 (38.1%) resided in Ortahisar, followed by Akçaabat (*n* = 213, 14.3%), Maçka (*n* = 194, 13.0%), of (*n* = 192, 12.9%), Yomra (*n* = 182, 12.1%), and Vakfıkebir (*n* = 145, 9.7%). Regarding occupational distribution according to the ISCO-08 classification, 420 (28.1%) participants were unemployed (including housewives and students), 278 (18.6%) were service and sales workers, 212 (14.2%) were retired, and 168 (11.3%) were professionals. Other categories included clerical support workers (*n* = 120, 8.0%), craft and related trades workers (*n* = 82, 5.5%), technicians and associate professionals (*n* = 76, 5.1%), elementary occupations (*n* = 61, 4.1%), managers (*n* = 37, 2.5%), plant and machine operators (*n* = 22, 1.5%), armed forces occupations (*n* = 10, 0.7%), and skilled agricultural, forestry, and fishery workers (*n* = 5, 0.3%). Regarding the health profiles, 955 (64.1%) of the participants perceived their health status favorably, with 15.2% reporting “Excellent” and 48.9% reporting “Good” health. In terms of healthcare facility preference, hospitals were the most frequently visited institutions (57.7%), followed by primary care centers (40.8%). Socio-demographic characteristics, health status and healthcare utilization characteristics of the participants are presented in Table 2.

**Table 2:** Socio-demographic characteristics, health status and healthcare utilization of the participants.

Characteristics	*n*	%
Gender		
Male	733	49.2
Female	758	50.8
Marital status		
Married	1,013	67.9
Single	478	32.1
Education level		
Illiterate/literate	61	4.1
Elementary school	246	16.5
Middle school	127	8.5
High school	540	36.2
University	461	30.9
Master’s/doctoral degree	56	3.8
Occupational status		
Has a profession	969	65.0
Has no profession	522	35.0
Employment status		
Employed	861	57.7
Unemployed	630	42.3
Insurance status		
Uninsured	126	8.5
Insured	1,365	91.5
Total household income (based on the national hunger limit)		
<1× HT	329	22.1
≥1× HT, <1.5× HT	292	19.6
≥1.5× HT, <2× HT	320	21.5
≥2× HT, <3× HT	244	16.4
≥3× HT	306	20.5
Self-rated health		
Excellent	226	15.2
Good	729	48.9
Fair	468	31.4
Poor	59	4.0
Very poor	9	0.6
Presence of chronic disease		
Yes	688	46.1
No	803	53.9
Prescribed chronic medication use		
Yes	654	43.9
No	836	56.1
Most frequently visited facility		
Primary care center	609	40.8
Hospital	861	57.7
Private clinic	21	1.4

HT, hunger threshold.

The mean score for perceived access to healthcare was 3.64 ± 0.64, with the highest scores in Accessibility (3.93 ± 0.76) and the lowest in Affordability (2.94 ± 1.04). The TR-HLS-EU-Q6 mean score was 2.72 ± 0.42. Regarding the Health Seeking Behavior Scale (3.12 ± 0.59), participants reported the highest inclination toward Professional health search (4.14 ± 0.68) and the lowest toward Online health search (2.57 ± 0.90). Detailed descriptive statistics for all scales and sub-dimensions are presented in Table 3.

**Table 3:** Descriptive statistics of the scales and sub-dimensions.

Scales and sub-dimensions	Mean ± SD	Min.–max
Perceived access to healthcare	3.64 ± 0.64	1.00–5.00
Acceptability	3.62 ± 0.78	1.00–5.00
Accessibility	3.93 ± 0.76	1.00–5.00
Affordability	2.94 ± 1.04	1.00–5.00
Accommodation	3.79 ± 0.68	1.00–5.00
TR-HLS-EU-Q6	2.72 ± 0.42	1.33–4.00
Health Seeking Behavior Scale	3.12 ± 0.59	1.00–5.00
Online health search	2.57 ± 0.90	1.00–5.00
Professional health search	4.14 ± 0.68	1.00–5.00
Search for traditional health	3.21 ± 0.95	1.00–5.00

TR-HLS-EU-Q6: Turkish version of Health Literacy Survey European-6 Item Short Form.

### 3.2 Spatial and linear regression analyses of factors influencing perceived access to healthcare

The relationship between the PAH dimensions and the independent variables was analyzed using both standard Ordinary Least Squares (OLS) and various spatial weight matrices (1 km, 3 km, 10 km, and Queen contiguity). While all OLS models demonstrated statistical significance (*p* < 0.001), the PAH Overall model yielded the highest explanatory power with an *R*^2^ value of 0.178. Regarding spatial dependence, the PAH Accessibility dimension exhibited a significant and positive spatial autoregressive coefficient within the 1 km distance band matrix (*ρ* = 0.315, *p* < 0.001). Conversely, no statistically significant spatial lag effect was detected for the PAH Overall, Acceptability, Accommodation, and Affordability dimensions when using 1 km and Queen contiguity matrices, as indicated by the Likelihood Ratio (LR) test results (*p* > 0.05). Furthermore, models utilizing 3 km and 10 km fixed distance bands were excluded from the final evaluation due to convergence failures, which prevented the calculation of log-likelihood values. Comprehensive coefficients, significance levels, and model fitness statistics for these analyses are detailed in Table 4.

**Table 4:** Table of comprehensive regression results for all models.

Dependent variable	Model/matrix	Spatial autoregressive coefficient *ρ* (Rho)/*λ* (Lambda)*	*R*-Squared ( $R^{2}$ )	*F*-statistic	*p*-value
PAH overall	OLS	–	0.178	40.025	<0.001
	Spatial lag (1 km)	−0.002	0.178	–	0.937
	Spatial lag (3 km)	Not Available (N/A)	N/A	–	–
	Spatial lag (10 km)	N/A	N/A	–	–
	Spatial lag (queen contiguity)	−0.119	0.178	–	0.323
PAH accessibility	OLS	–	0.140	30.023	<0.001
	Spatial error (1 km)	0.315*	0.152	–	<0.001
	Spatial lag (3 km)	N/A	N/A	–	–
	Spatial lag (10 km)	N/A	N/A	–	–
	Spatial lag (queen contiguity)	0.047	0.140	–	0.656
PAH acceptability	OLS	–	0.139	29.795	<0.001
	Spatial lag (1 km)	−0.013	0.139	–	0.684
	Spatial lag (3 km)	N/A	N/A	–	–
	Spatial lag (10 km)	N/A	N/A	–	–
	Spatial lag (queen contiguity)	−0.162	0.140	–	0.215
PAH accommodation	OLS	–	0.172	38.434	<0.001
	Spatial lag (1 km)	−0.020	0.172	–	0.479
	Spatial lag (3 km)	N/A	N/A	–	–
	Spatial lag (10 km)	N/A	N/A	–	–
	Spatial lag (queen contiguity)	−0.192	0.174	–	0.146
PAH affordability	OLS	–	0.050	9.827	<0.001
	Spatial lag (1 km)	−0.016	0.050	–	0.763
	Spatial lag (3 km)	N/A	N/A	–	–
	Spatial lag (10 km)	N/A	N/A	–	–
	Spatial lag (queen contiguity)	−0.203	0.174	–	0.112

Models utilizing 3 km and 10 km fixed distance band matrices were excluded from the analysis as they failed to converge and their log-likelihood values could not be computed.

The *p*-value column represents probability (*F*-statistic) for OLS models and the Likelihood Ratio Test (LR Test) probability for spatial models.

The results of the Spatial Error Model, utilizing a 1 km distance band to identify the factors influencing perceived access to healthcare (PAH Accessibility) while controlling for age and gender, are presented in Table 5. Upon examining the model statistics, the *R*^2^ value of 0.152 and the statistically significant spatial error coefficient [Lambda (*λ*) = 0.315, *p* < 0.001] indicate that the model successfully accounts for the spatial dependence in the data, providing more robust estimations than the standard OLS model. When demographic factors are held constant, Health Literacy (*β* = 0.269, *p* < 0.001), Professional Health Search Behavior (*β* = 0.319, *p* < 0.001), and self-rated health (*β* = 0.074, *p* = 0.002) emerge as the strongest significant positive predictors of accessibility perceptions. Conversely, Online Health Search Behavior (*β* = −0.080, *p* < 0.001) has a significant negative impact on perceived accessibility. Regarding the control variables, gender was found to have a significant effect on accessibility perceptions (*β* = −0.080, *p* = 0.032). Based on the coding scheme (Male = 1, Female = 2), this negative coefficient reveals that women have lower perceived access to healthcare compared to men. No statistically significant effects were observed for age, the socioeconomic status (SES) index, or Traditional Health Information Seeking on perceived “accessibility” (*p* > 0.05).

**Table 5:** Spatial Error Model (SEM) results for perceived accessibility to healthcare (1 km distance band).

Variable	PAH accessibility (1 km distance band)	
	Coefficient (*B*)	*p*
Spatial error (lambda)	0.315	**<0.001**
Constant	1.821	**<0.001**
SES index	0.002	0.618
Health literacy	0.269	**<0.001**
Online health search	−0.080	**<0.001**
Professional health search	0.319	**<0.001**
Traditional health search	−0.006	0.739
Self-rated health	0.074	**0.002**
Control variables		
Age	0.002	0.123
Gender	−0.080	**0.032**
Model statistics		
Observations (*N*)	1,491	–
*R*-squared (*R*^2^)	0.152	–
AIC	3199.1	–
Log-likelihood	−1590.54	–

The *p*-values in bold indicate statistical significance at the *p* < 0.05 level.

### 3.3 Structural Equation Modeling and mediation analysis of perceived access to healthcare

**Figure 5:**
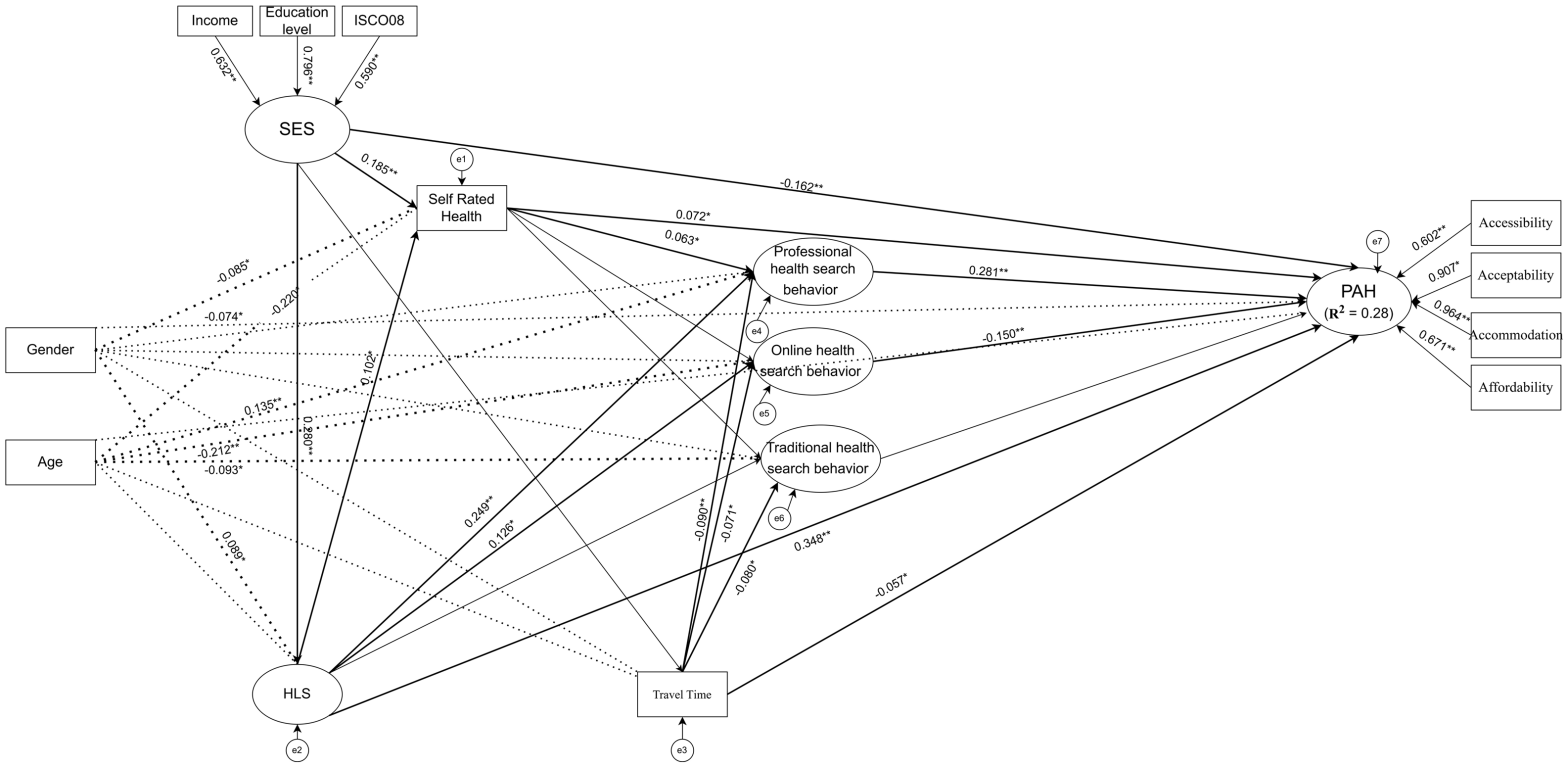
An investigation of factors influencing perceived access to healthcare using Structural Equation Modeling (SEM). For clarity, only statistically significant paths (*p* < 0.05) are displayed. Hypothesized main effects are represented by bold arrows, while control variables (Age, Gender) are indicated by dashed lines. Values on the paths represent standardized path coefficients (beta). SES, socio-economic status, HLS, health literacy scale, PAH, perceived access to healthcare ^*^p < 0.05, ^**^p < 0.001.

The fit of the structural model was evaluated using several goodness-of-fit indices. The results indicated a satisfactory fit to the data: chi^2^/df = 3.923, RMSEA = 0.044 (90% CI: 0.043–0.046), GFI = 0.897, CFI = 0.891, TLI = 0.879 and SRMR = 0.0549. An RMSEA value below 0.05 indicates an excellent fit. Although the CFI and TLI values were slightly below the conventional 0.90 threshold, they are considered acceptable given the large sample size (*N* = 1,491) and the complexity of the structural model. The final structural model, including standardized path coefficients and their significance levels, is presented in Figure 5.

The analysis revealed that PAH scores remained statistically invariant across all evaluated socioeconomic parameters, including household income, educational attainment, and occupational groups (*p* > 0.05). Conversely, a significant upward gradient was observed for both TR-HLS-EU-Q6 and self-rated health scores in relation to increasing income and educational levels (*p* < 0.001). Furthermore, occupational categories significantly influenced health literacy (*p* = 0.003) and self-rated health (*p* < 0.001), with the highest mean values consistently reported by managers and professionals, while the lowest scores were observed among the retired and unemployed cohorts. Detailed inter-group comparisons and associated *p*-values are summarized in Table 6.

**Table 6:** Comparison of PAH, health literacy, and self-rated health scores by socio-economic characteristics.

Characteristics	PAH (Mean ± SD)	TR-HLS-EU-Q6 (Mean ± SD)	Self-rated health (Mean ± SD)
Household income level			
<Hunger limit	3.63 ± 0.68	2.60 ± 0.38	3.46 ± 0.85
1–2 times hunger limit	3.64 ± 0.64	2.77 ± 0.41	3.63 ± 0.71
2–3 times hunger limit	3.67 ± 0.64	2.72 ± 0.43	3.87 ± 0.75
3–4 times hunger limit	3.67 ± 0.60	2.75 ± 0.43	3.86 ± 0.73
>4 times hunger limit	3.61 ± 0.63	2.77 ± 0.44	3.92 ± 0.75
*p*	0.674	**<0.001**	**<0.001**
Educational attainment			
Literate/no degree	3.69 ± 0.62	2.52 ± 0.43	3.13 ± 0.81
Primary school	3.72 ± 0.62	2.63 ± 0.40	3.42 ± 0.80
Middle school	3.67 ± 0.65	2.71 ± 0.43	3.58 ± 0.83
High school	3.62 ± 0.67	2.71 ± 0.40	3.81 ± 0.74
University degree	3.61 ± 0.63	2.79 ± 0.43	3.92 ± 0.71
Postgraduate degree	3.74 ± 0.58	2.79 ± 0.50	3.98 ± 0.73
*p*	0.176	**<0.001**	**<0.001**
Occupational group			
Not employed	3.69 ± 0.57	2.73 ± 0.43	3.62 ± 0.78
Retired	3.67 ± 0.67	2.67 ± 0.44	3.48 ± 0.80
Operators and laborers	3.66 ± 0.62	2.60 ± 0.37	3.76 ± 0.64
Agriculture and crafts	3.69 ± 0.63	2.77 ± 0.47	3.80 ± 0.96
Clerical, service, and sales	3.59 ± 0.70	2.71 ± 0.37	3.84 ± 0.75
Managers and professionals	3.62 ± 0.63	2.79 ± 0.49	3.97 ± 0.70
*p*	0.193	**0.003**	**<0.001**

PAH, perceived access to healthcare; TR-HLS-EU-Q6: Turkish version of Health Literacy Survey European-6 Item Short Form; SD, standard deviation.

The *p*-values in bold indicate statistical significance at the *p* < 0.05 level.

The analysis of direct and indirect effects revealed complex mediation pathways within the structural model. Specifically, socioeconomic status (SES) exhibited a significant negative direct effect on the dependent variable (*β* = −0.162, *p* < 0.001). Conversely, the indirect effect of SES through the mediating variables was positive and significant (*β* = 0.135, *p* < 0.001). Due to the opposing directions of the direct and indirect paths, the total effect of SES became non-significant (*β* = −0.027, *p* = 0.515).

Health Literacy (HL) emerged as the most potent predictor in the model. HL significantly and positively predicted the dependent variable through both direct (*β* = 0.348, *p* = 0.001) and indirect pathways (*β* = 0.061, *p* = 0.001), resulting in a substantial total effect size of *β* = 0.408 (*p* < 0.001).

Regarding self-rated health, a significant positive total effect was observed (*β* = 0.089, *p* = 0.004). This influence was primarily driven by the direct path (*β* = 0.072, *p* = 0.019), whereas the indirect effect did not reach statistical significance (*p* = 0.101).

Finally, the Travel Time score demonstrated a significant negative total effect (*β* = −0.075, *p* = 0.004). Both the direct effect (*β* = −0.057, *p* = 0.024) and the indirect effect (*β* = −0.018, *p* = 0.053) contributed to this outcome, with the direct path being the primary driver of statistical significance. The decomposition of standardized direct, indirect, and total effects is summarized in Table 7.

**Table 7:** Standardized direct, indirect, and total effects of the structural model.

Variables	Standardized total effect		Standardized direct effect		Standardized indirect effect	
	*β*	*p*	*β*	*p*	*β*	*p*
SES index	−0.027	0.515	−0.162	**<0.001**	0.135	**<0.001**
TR-HLS-EU-Q6	0.408	**<0.001**	0.348	**0.001**	0.061	**0.001**
Self rated health	0.089	**0.004**	0.072	**0.019**	−0.017	0.101
Travel time	−0.075	**0.004**	−0.057	**0.024**	−0.018	0.053

SES, socioeconomic status; TR-HLS-EU-Q6, Turkish version of Health Literacy Survey European-6 Item Short Form.

The *p*-values in bold indicate statistical significance at the *p* < 0.05 level.

To further investigate the specific mechanisms of these indirect effects, a bootstrapping analysis with 5,000 resamples was performed (Table 8). The analysis of specific indirect paths revealed that the relationship between SES and perceived access was primarily mediated through health literacy (*β* = 0.041, *p* < 0.001) and self-rated health (*β* = 0.006, *p* = 0.016).

**Table 8:** Standardized specific indirect effects of the structural model (Bootstrap: 5000 samples).

Mediation path	*β*	95% BC CI [Lower. Upper]	*p*
SES-driven paths			
SES ➔HLS➔PAH	0.041	[0.026, 0.060]	**<0.001**
SES ➔ self rated health➔ PAH	0.006	[0.001, 0.012]	**0.016**
SES ➔travel time➔ PAH	0.001	[0.000, 0.005]	0.064
Health literacy-driven paths			
HLS ➔online health search➔ PAH	−0.042	[−0.101, −0.010]	**0.004**
HLS ➔professional health search➔ PAH	0.154	[0.093, 0.248]	**<0.001**
HLS ➔traditional health search➔ PAH	0.001	[−0.008, 0.017]	0.628
HLS ➔self rated health➔ PAH	0.016	[0.003, 0.041]	**0.010**
Self-rated health-driven paths			
Self rated health ➔ online health search ➔ PAH	−0.001	[−0.007, 0.005]	0.756
Self rated health ➔ professional health search ➔ PAH	0.011	[0.000, 0.024]	**0.041**
Self rated health ➔ traditional health search ➔ PAH	0.001	[−0.001, 0.006]	0.317
Travel time-driven paths			
Travel time ➔ online health search➔ PAH	0.012	[−0.003, 0.025]	**0.006**
Travel time ➔ professional health search➔ PAH	−0.028	[−0.050, −0.013]	**<0.001**
Travel time ➔ traditional health search➔ PAH	−0.004	[−0.014, 0.002]	0.190

SES, socioeconomic status; HLS, health literacy scale; PAH, perceived access to health care; BC CI, bias-corrected confidence interval.

The *p*-values in bold indicate statistical significance at the *p* < 0.05 level.

## 4 Discussion

Since its establishment as a developing country in 1923, the Republic of Türkiye has adopted various models of health organization. Long before the 1978 Alma-Ata Declaration, it established the principle of delivering services at the location closest to where the individual lives. This principle was initially implemented through the “government medical officer” system, followed by the “health center” system, and is currently embodied by the “family medicine” system (58, 59). However, as a consequence of neoliberal policies, the concentration of profit-driven diagnostic and treatment services in specific centers and metropolitan areas has driven healthcare toward a more costly and centralized structure, creating a structural shift (11, 60). Moreover, income disparities, exacerbated by economic and political crises at both global and national scales, have led to inequities among different segments of society, thereby triggering health inequalities (61). This study was designed with the fundamental motivations outlined above in mind. It addresses a significant gap in the literature by analyzing factors believed to influence health access. These factors include socioeconomic status, health literacy, health-seeking behavior, self-rated health, and travel time. The analysis uses Structural Equation Modeling (SEM) and spatial modeling. The study offers significant insights into the perception of access to health services by the public and the factors influencing it in Trabzon, a city with metropolitan status.

### 4.1 Spatial and linear regression analyses of factors influencing perceived access to healthcare

In the present study, spatial analyses focused on both the overall perceived access to healthcare (PAH Overall) scores and the sub-dimensions of the scale. Among the various models tested, the OLS model established for PAH overall was identified as the model with the highest explanatory power for the variance in the dependent variable. While the findings of the spatial regression models indicated no spatial dependence for PAH Overall across different weight matrices (1 km, Queen contiguity), a significant spatial dependence was detected for the PAH Accessibility sub-dimension within the 1 km distance band. Furthermore, the failure of the models to converge at broader scales (3 km and 10 km) suggests that the spatial interaction regarding healthcare access in this region is highly localized in nature. This localized distribution demonstrates that individuals’ perceptions are positively influenced by their neighborhood experiences within a narrow radius of approximately 1 km. Trabzon Province is distinguished by its mountainous topography, characterised by a series of mountains that extend from east to west across the southern portion of the province. These mountains are interspersed with a narrow coastal strip along the Black Sea coast and extensive plateaus that have been sculpted into profound valleys by the rivers that traverse these landscapes. A geographical analysis of the province reveals that 30% of its total area is mountainous, 60% consists of regions where the slope increases to 25–30% towards the south, and a mere 10% consists of flat areas. This phenomenon has resulted in the establishment of settlements on steep slopes (41). From this standpoint, the results of our analysis suggest that, within a city such as Trabzon, where topographic barriers are substantial, individuals residing within the same 1 km radius are subject to comparable physical constraints.

### 4.2 The impact of health literacy on perceived access to healthcare

In Structural Equation Modeling (SEM), a methodology employed to explore the dynamics of perceived access to healthcare, health literacy (HL) appears to play a significant role, both through its direct effect and via mediating pathways. The research findings indicate that HL is the variable with the strongest total effect size on perceived access to health services. According to Andersen’s Health Behavior Model, HL functions as a facilitator; that is, as an individual’s capacity to understand and evaluate health information increases, they perceive the systemic complexity of accessing health services as less of a barrier (5). In this study, one of the primary mechanisms through which health literacy enhances access is by triggering professional health-seeking behaviors. The results of the bootstrapping analysis demonstrated that, while health literacy significantly enhances individuals’ perception of access to healthcare through professional health-seeking channels, it concomitantly exerts a negative influence on online health-seeking behavior. The study further revealed that health literacy does not exert any discernible influence on traditional health-seeking behavior. In a similar vein, the study by Akakpo and Neurer (62) found that an enhancement in health literacy levels was associated with an increase in professional health-seeking behaviors, such as seeking care at a healthcare facility when experiencing a health issue. The study concluded that there was no effect on traditional methods, including self-medication and the use of herbal remedies. Consequently, this noteworthy role of health literacy in access demonstrates that access to the healthcare system is not merely a matter of physical distance or socioeconomic ability, but also involves the cognitive ability to understand and utilise the system. Furthermore, the findings reveal that health literacy functions as a guiding compass in accessing the healthcare system, serving as a filter that steers individuals away from traditional or digital uncertainties and directs them towards professional healthcare-seeking behavior. This finding provides scientific validation for the necessity of educational interventions aimed at enhancing the individual’s “navigation capacity” within the system, which should be accorded a central role in health policies.

### 4.3 The impact of health seeking behavior on perceived access to healthcare services

The structural equation model suggests that individuals’ methods of seeking solutions to health problems have opposing associations with perceived access. The behaviors of “professional health search” and “online health search” play a significant mediating role in the model. These behaviors are related to perceived access to health services in opposite directions. The finding that professional health-seeking behavior emerged as one of the strongest positive predictors of perceived access in the study is particularly noteworthy, given that a significant portion of health literacy’s indirect effect on access occurs through this channel. This observation underscores the importance of the connection established with the healthcare system. An individual’s propensity to address health concerns in strict compliance with a physician’s directives has been demonstrated to positively impact the quality of communication within the healthcare system (63). One of the study’s interesting findings is that online health-seeking behavior has a negative association with perceived access. Contrary to the prevailing notion that digitalization enhances access, this study posits that the digital information-seeking process serves to augment “perceived barriers.”

### 4.4 The impact of socioeconomic status on perceived access to healthcare

A salient finding from the Structural Equation Modeling (SEM) analysis pertains to the complex effects of socioeconomic status (SES) on access. The analysis results indicated that SES has a significant negative direct effect on the dependent variable within this model; however, it exerts a positive indirect effect on access through health literacy (HL) and self-rated health. Despite the plethora of studies in various societies that have been conducted in the extant literature arguing for a positive correlation between high socioeconomic status and easier access to healthcare, the “inconsistent mediation” relationship identified in this study points to a more profound underlying mechanism (64–66).

The direct negative effect identified in the study can be explained by the “expectation and adaptation” mechanism highlighted by Gulliford et al. (67) and Lakin and Kane (68). While individuals with limited access to services or constrained living conditions gradually adapt by optimizing their expectations regarding the service (adaptation), the standards of individuals with high socioeconomic status regarding service quality, speed, and delivery are rising. This phenomenon prompts individuals, despite having objective access to the service, to subject it to a more critical evaluation in their subjective assessment, thereby leading to a decline in their perception of access. As emphasized in the study by Willems et al. (69), the fact that patients in the high SES group demand more information from the physician and expect more active participation in the process confirms this group’s rising standards and critical approach toward the service.

The findings from the bootstrapping analysis suggest that the indirect impact of SES on perceptions of access is mediated through both cognitive and physical channels. In Andersen’s (5) model, SES is defined as an “enabling factor”. In this context, high SES facilitates the overcoming of structural barriers by enabling individuals to better analyze the health system and evaluate their own health status more positively (70–72). Indeed, the findings of this study align with field observations suggesting that individuals with higher socioeconomic status do not limit their health-seeking behaviors to local resources. This group exhibits a tendency to seek high-level referral centers in major metropolises, such as Istanbul, for specialized treatment processes requiring advanced expertise. In light of these data, it is hypothesized that the relatively positive claims regarding healthcare access made by individuals with lower socioeconomic status may, in fact, reflect a ‘lowered expectation level’ shaped by limited transportation options, insufficient health awareness, and low health literacy. In other words, the perceived access levels of these individuals may be masked by a psychological adaptation mechanism developed to compensate for their existing systemic constraints.

### 4.5 The impact of travel time on perceived access to healthcare services

In this study, travel time was identified as a critical physical barrier that exerts a significant and negative influence on the perceived access to healthcare among the participants. This impact is primarily driven by a direct pathway. This finding indicates that as the time required to reach a healthcare facility increases, individuals’ overall perception of accessibility tends to weaken, emphasizing that physical distance remains a relevant factor in healthcare utilization. This result is consistent with the literature demonstrating the negative impact of increased access time on the utilization of health services (73, 74).

The effect of travel time is more clearly understood through its interaction with health-seeking behaviors. Bootstrapping analysis revealed that travel durations exceeding 15 min negatively affect access perception by decreasing Professional Health-Seeking Behavior. This suggests that prolonged travel durations are not merely a logistical challenge but also a deterrent that discourages individuals from seeking professional help, potentially leading to delays in treatment processes. Interestingly, a small but significant positive indirect effect was observed through Online Health-Seeking Behavior. This indicates that when physical barriers (travel time) increase, individuals turn to digital health resources to compensate for the difficulties in physical access; however, this orientation does not completely eliminate the negative impact created by distance. Considering Trabzon’s mountainous topography and dispersed settlement pattern, these results gain particular importance. Although Family Health Centers (FHCs) are strategically distributed at the neighborhood level, the findings suggest that the time required to reach healthcare institutions continues to be a systemic obstacle.

### 4.6 Limitations and strengths

Several limitations should be acknowledged when interpreting the findings of this study. First, the study utilized a cross-sectional design, which precludes the establishment of definitive causal relationships between perceived access to healthcare with other determinants. Second, beyond the variables incorporated into the Structural Equation Modeling (SEM) framework, there may be other individual and societal factors defined in the literature that influence perceived access to healthcare. Third, a specific methodological choice was made regarding travel time calculations. Although a wide range of transit modes—including public transportation—was recorded during the survey, public transport usage was analyzed using the “driving car” mode in ORS tools. Utilizing this proxy may underestimate the total travel time for transit users by excluding waiting durations at stops and the time required for pedestrian access to transit points. Furthermore, since the data collected from participants was based on self-reports via a single survey instrument, there is a possibility of common-method bias and recall bias. Finally, while these findings provide important insights for the study area, caution should be exercised when generalizing the results to other geographic or policy contexts with different healthcare infrastructure.

Despite these limitations, the study possesses significant strengths. The use of a large-scale dataset collected through face-to-face interviews with 1,491 participants enhances the statistical robustness of the findings. Moreover, the integration of geospatial data (GIS) with comprehensive survey instruments allowed for a multidimensional analysis of numerous variables. This approach has led to critical insights into the determinants of healthcare access, making a substantial contribution to the existing body of knowledge.

## 5 Conclusion

Structural Equation Modeling (SEM) confirms that health literacy and professional health search behavior are the primary positive drivers in determining perceived access to healthcare. In contrast, online health searching and increased travel time emerge as factors that diminish the perception of accessibility. The direct negative impact of socioeconomic status (SES) on access is balanced through mediation mechanisms, notably health literacy and self-rated health status. This suggests that the disadvantages posed by low SES can be overcome by enhancing health education and literacy capacity. Considering that individuals with high SES tend to be more critical of existing services due to higher expectations, while those with lower SES tend to adapt to local resources, the provision of inclusive and high-quality local services remains of critical importance. Consequently, integrating sustainable approaches that improve health literacy with modern interventions aimed at minimizing physical barriers, such as travel time, will contribute significantly to reducing health inequalities and improving the overall public perception of access.

Although there is no significant spatial relationship regarding total perceived access to healthcare scores, distinct spatial dependencies appear within the ‘accessibility’ sub-dimension at a one-kilometre distance band. This indicates that individuals’ general perceptions of healthcare access are primarily shaped by personal factors, independent of spatial boundaries. However, their perceptions of accessibility seem to be more directly influenced by geographical location and the local environment. These findings are considered to provide a scientific basis for health planning in challenging topographies such as Trabzon, shifting the focus from macro-scale strategies to micro-spatial interventions aimed at minimising physical barriers at neighbourhood and street levels. In this context, strategically placing primary healthcare services in highly accessible locations is thought to be essential to ensure equitable access and enhance the perceived level of service availability in emerging residential areas.

## Data Availability

The raw data supporting the conclusions of this article will be made available by the authors, without undue reservation.
