# Supplementary material for: What influences the perceived access to healthcare services? An investigation using Structural Equation Modeling
Source: Front Public Health. 2026 Jun 4;14:1844139. doi: 10.3389/fpubh.2026.1844139 (PMC13275417; doi:10.3389/fpubh.2026.1844139)
Supplement: Supplementary file 1 [file Supplementary_File_1.pdf]

Supplementary Material

Methodological Note on Validity and Reliability Analyses:

Since all scales utilized in this study (Turkish Version of European Health Literacy Survey Short Form, Perceived Access to Healthcare Services, and Health Seeking Behavior) are previously validated instruments, Confirmatory Factor Analysis (CFA) was directly conducted to verify their structural validity within the current study population. Analyses were performed using IBM SPSS AMOS (Version 24.0) software. Model fit was assessed using the ratio of Chi-square to degrees of freedom (chi^2/df), Comparative Fit Index (CFI), Tucker-Lewis Index (TLI), and Root Mean Square Error of Approximation (RMSEA). Internal consistency was confirmed using Cronbach's Alpha coefficients at both the sub-dimension and total scale levels.

Supplementary Table 1. Characteristics of the Utilized Scales

**Scale**	**Sub-dimensions**	**Number of Items**	**Source**
Perceived Access to Healthcare Scale	Accessibility (4 Items)<br>Acceptability (8 Items)<br>Accommodation (8 Items)<br>Affordability (3 Items)	23	Yilmaz et al., 2025 (49)
Turkish Version of European Health Literacy Survey Short Form (TR-HLS-EU-Q6)	None	6	Yesildal, 2025 (46)
Health Seeking Behavior	Online Health Search (6 Items)<br>Professional Health Search (3 Items)<br>Traditional Health Search (3 Items)	12	Kirac and Ozgür, 2021 (47)
SES (Socioeconomic status) Index	None	3	Developed by the authors*

**The Socioeconomic Status (SES) Index was developed by the authors for the purpose of this study, integrating three key objective indicators: educational level, monthly household income, and occupational status. These variables were combined to create a composite score representing the socioeconomic standing of the participants.*

Supplementary Table 2. Construct Validity and Reliability Results (CFA Results)

**Scale and Subdimensions**	**Item Code**		**Standardized Factor Loadings (λ)**	**Cronbach's α**
**Perceived Access to Healthcare Scale**				0.929
Accessibility	Item 1	The services I need are provided at the health center.	0,518	0.825
	Item 2	The distance from the health centers to my house is appropriate.	0,814	
	Item 3	The time required to reach the health center is appropriate.	0,836	
	Item 4	Getting to and from the health center is easy for me.	0,881	
Acceptability	Item 5	The health services (immunization, medical visit, family planning, mother and childcare, injections, etc.) I need are provided at the public health center.	0,688	0.892
	Item 6	Health staff is tailored to the number of clients and their needs.	0,765	
	Item 7	The quality of services provided in the health center is acceptable.	0,754	
	Item 8	The health center staff meets the needs of the clients in various ways, such as being introduced to the community resources.	0,756	
	Item 9	Health workers listen carefully to what I have to say.	0,757	

	Item 10	The health workers give me enough time.	0,711	
	Item 11	I trust the statements of the treatment team (doctor, nurse, midwife, etc.)<br><br>about my health and illness.	0,717	
	Item 12	My request for same sex health care professionals is taken into account.	0,497	
Accommodation	Item 13	The treatment team at the health center is respectful.	0,687	0.860
	Item 14	The working hours of the public health center are suitable for receiving<br><br>services from these centers.	0,61	
	Item 15	The physical space of the health center is suitable for receiving services.	0,627	
	Item 16	Access to some facilities such as wheelchairs, walkers, etc. is provided<br><br>in the health center.	0,463	
	Item 17	The educations that are given to me are such that I understand them.	0,623	
	Item 18	The information I need is expressed in simple language without the use<br><br>of specialized words.	0,755	
	Item 19	Communication of health workers (doctor, nurse, midwife, etc.) with<br><br>clients is appropriate.	0,716	
	Item 20	Health workers try to make sure I fully understand the health<br><br>information provided.	0,686	

Affordability	Item 21	It is easy to make an appointment at a health center.	0,569	0.769
	Item 22	The expected time to receive the services I need is appropriate.	0,641	
	Item 23	My living conditions are taken into accounts, such as marital status,<br><br>ability to pay, and cultural differences.	0,753	
**TR-HLS-EU-Q6**				
	Item 1	Judge when you may need to get a second opinion from another doctor	0.329	0.662
	Item 2	Use information the doctor gives you to make decisions about your illness	0.456	
	Item 3	Find information on how to manage mental health problems like stress or depression	0.719	
	Item 4	Judge if the information on health risks in the media is reliable	0.319	
	Item 5	Find out about activities that are good for your mental well-being	0.556	
	Item 6	Understand information in the media on how to get healthier	0.395	
**Health Seeking Behavior**				0.720
Online Health Search	Item 1	I do research on the internet about my disease	0,452	0.772
	Item 2	I follow programs about my illness on television	0,427	
	Item 3	I contact the doctors on the internet about my disease	0,589	

	Item 4	I look at the side effects of the drugs I use on the Internet when I get sick	0,551	
	Item 5	I follow the forms about my illness on the Internet	0,808	
	Item 6	I scan in journals and books about my disease	0,742	
Professional	Item 7	When I am ill, I apply to the physician immediately	0,681	0.781
	Item 8	I pay attention to the advise of physician about my disease	0,896	
	Item 9	I try to take the food recommended by the doctor	0,688	
Traditional	Item 10	I try to heal with herbal drugs at home	0,380	0.654
	Item 11	I pay attention to the advice of people I trust for my illness	0,758	
	Item 12	I pay attention to the advice of people who have had the same disease before	0,729	
**Socioeconomic Status (SES) Index**				0.692
	Item 1	Monthly household income	0.691	
	Item 2	Education level	0.731	
	Item 3	Occupational status	0.610	

Supplementary Table 3. Confirmatory Factor Analysis (CFA) Goodness-of-Fit Indices for the Utilized Scales

Fit Index	Perceived Access to Healthcare	TR-HLS-EU-Q6	Health Seeking Behavior
**χ2/df**	7.608	5.790	4.936

**CFI**	0.921	0.978	0.959
**TLI**	0.906	0.945	0.946
**RMSEA**	0.067	0.057	0.051
